# Positive Feedback Regulation of Proliferation in Vascular Smooth Muscle Cells Stimulated by Lipopolysaccharide Is Mediated through the TLR 4/Rac1/Akt Pathway

**DOI:** 10.1371/journal.pone.0092398

**Published:** 2014-03-25

**Authors:** Dehua Jiang, Dongye Li, Lijuan Cao, Lele Wang, Shasha Zhu, Tongda Xu, Cheng Wang, Defeng Pan

**Affiliations:** 1 Institute of Cardiovascular Disease Research, Xuzhou Medical College, Xuzhou, Jiangsu, China; 2 Affiliated Hospital of Xuzhou Medical College, Xuzhou, Jiangsu, China; IISER-TVM, India

## Abstract

Toll-like receptor 4 (TLR4) are important in inflammation and regulating vascular smooth muscle cells (VSMCs) proliferation, which are related to atherosclerosis and restenosis. We have investigated the mechanisms involved in Lipopolysaccharide (LPS)-induced proliferation of VSMCs. Stimulation of rat aortic VSMCs with LPS significantly increases the proliferation of VSMCs. This effect is regulated by Rac1 (Ras-related C3 botulinum toxin substrate l), which mediates the activation of phosphatidylinositol 3-kinase/Akt (PI3K/Akt) signaling pathways. Inhibition of Rac1 activity by NSC23766 is associated with inhibition of Akt activity. Treatment with NSC23766 or LY294002 significantly decreases LPS-induced TLR4 protein and mRNA expression. The data show that positive feedback regulation of proliferation in VSMCs is mediated through the TLR4/Rac1/Akt pathway.

## Introduction

Abnormal proliferation of VSMCs are centrally involved in the pathogenesis of early pathological vascular hyperplasic lesions, such as atherosclerosis and restenosis, after percutaneous coronary intervention (PCI) and in the development of hypertension [1], [2]. Finding the gene that regulates cell proliferation would help in preventing vascular hyperplastic disease.

Toll-like receptors (TLRs) are one of pathogen pattern recognition receptors. They can activate the regulation of innate and adaptive immunity involved in the inflammatory response [3]. TLR4, an important member of the TLRs families, may be intimately involved in the development and incidence of vascular hyperplastic lesions [4]. Epidemiological research suggests that LPS is a strong risk factor for cardiovascular disorders [5]. Previous studies have also demonstrated that TLR4 is abundantly expressed in the surface of macrophages, neutrophils, and dendritic cells [6]. An association between the functional expression of TLR4 and the subsequent augmentation of intimal hyperplasia has been described [7]. TLR4 exists in VSMCs, and may be implicated in restenosis, and therefore may make a fundamentally significant contribution to the crucial pathophysiological relationship between inflammation and cardiovascular disorders [8]. LPS-induced systemic inflammatory responses could exacerbate neointima formation after balloon injury and stent implantation, with the resulting proliferation of VSMCs playing a key role in atherogenesis.

PI3K is upstream of NF-κB pathway activation, and the NF-κB signal transduction pathway is the most important downstream pathways mediated by LPS signal pathways [9]. Rac1 participates in the LPS-mediated signal transduction [10]. TLR2 mediated NF-κB activation requires a Rac1-dependent pathway [11]. Whether Rac1 participates in LPS-induced TLR4 activation on regulation of VSMCs proliferation has not been reported.

LPS is considered to be a strong stimulator for the pathogenesis of atherosclerosis, and low concentrations of LPS induce potent inflammatory activation in intact human blood vessels. VSMCs also exhibit lower expression of TLR4 under basal conditions. LPS treatment can upregulate TLR4 expression and promote a pro-inflammatory phenotype in VSMCs, which may potentially be involved in vascular inflammation [12]. However, the function and mechanisms of how LPS affects VSMCs proliferation remain obscure.

## Materials and Methods

### Cell Culture

Thoracic aortas were resected from Sprague-Dawley rats (8–10 weeks old, male or female, 200±20 g, provided by the Institute of Laboratory Animal of Xuzhou Medical College). The experimental protocols particularly in respect to the ethical animal care and use of animals were approved by the ethical Committee in Xuzhou Medical College. Cells were used at passages 3 to 8. Primary VSMCs culture was prepared from the aortas using the explant technique. VSMCs were maintained in Dulbecco’s modified eagle medium (DMEM, Hyclone, USA) containing 10% heat-inactivated fetal bovine serum (FBS, Gibco/Invitrogen, USA), 100 U/ml penicillin, and 100 mg/ml streptomycin at 37°C under air with 5% CO2. The cells were serum-starved for 24 h before treatment or stimulation with reagents.

### Analysis of Cell Viability

Cell viability was measured by the MTT assay and cell counting. VSMCs were stimulated with LPS at from 0 to 100 μg/ml for 24 h before MTT solution was added at 0.5 mg/ml, and were incubated at 37°C for 1 h. After removing the medium, 150 ul DMSO was added and the optical density of each well read at 590 nm. For the cell counting assay, VSMCs (1×105/ml) were present in 12-well plates. After incubation with LY294002 (10 μmol/ml) 1 h or NSC23766 (25 μmol/m) 24 h separately, VSMCs were stimulated with 10 ug/ml LPS for 24 h and the number of cells in each well counted after treatment.

### 5-ethynyl-2′-deoxyuridine (EdU) Staining

EdU staining used Cell-LightTM EdU Kit (Rui Bo Guangzhou Biotechnology Limited Company, China) with modifications [13]. VSMCs in logarithmic growth phase were seeded in 6-well plates and stimulated with LPS (10 μg/ml) in the presence or absence of NSC23766 (25 μM) 24 h or LY294002 (10 μM) 1 h. Each well was treated with 500 μl EdU (50 μM) and incubated for 2 h. After washing in 2 mg/ml glycine solution diluted in double distilled water for 10 min, the sections were permeabilized with 0.5% Triton X-100 in PBS for 10 min, and washed with PBS for 5 min. Apollo reaction buffer liquid, catalyst, fluorescent dyes and buffer additives were dissolved in deionized water, and shaken to make the Apollo 567 staining reaction solution. The sections were incubated for 30 min in the dark, and washed twice with PBS for 10 min. For DNA staining, sections were counterstained with 500 ul 1X Hoechst 33342 for 30 min in the dark. The slides were washed twice with PBS for 3 min, and examined immediately under a fluorescence microscope (magnification ×400). All the procedures were done at room temperature. An Olympus BX51 microscope (Olympus, Japan) was used to detect EdU-positive cells. Images of the Apollo 567 were taken with a “red” filter set: excitation 550 nm, emission: 565 nm, filter: 555±15 nm. Images of the Hoechst 33342 were taken with a “blue” filter set: excitation: 350 nm, emission: 461 nm, filter: 405±15 nm.

### Western Blot Analysis

Western blot analysis was used to determine the levels of TLR4 and Rac1, and activation of p-Akt (308), p-Akt (473) in VSMCs stimulated with LPS (10 μg/ml, Sigma, USA; 98% purity). Twenty μg protein were resolved on 10 or 12% SDS-polyacrylamide gel electrophoresis (SDS-PAGE) and transferred to polyvinylidene fluoride (PVDF) membranes, which were blocked with 5% BSA. The PVDF membranes were probed with primary antibodies (Cell signaling), anti-TLR 4 antibody, anti-Rac1/2/3 antibody, anti-p-Akt (308) antibody, and anti-p-Akt (473) antibody, being incubated overnight at 4°C. The membranes were washed in PBS-0.1% Tween 20 and incubated with HRP-conjugated secondary antibodies for 1 h at room temperature. After successive washes, the membranes were developed using an ECL kit. Mouse anti-β-actin was used as a loading control. Protein expression levels were quantified as optical densities.

### Pull-Down Assay for Rac1 Activity

Rac1 activation was measured using a glutathione S-transferase (p21-activated kinase)–p21 binding domain (GST– [PAK]–PBD) fusion protein, which binds activated Rac1. VSMCs were lysed and centrifuged at 12,000×g for 10 min at 4°C. The supernatant was collected and incubated with GST–(PAK)–PBD fusion protein. The protein-bead complexes were recovered by centrifugation and washed. After successive washes, the protein-bead complexes were resuspended in SDS and resolved on 12% SDS–PAGE. The proteins were transferred to PVDF membrane and the membrane was incubated with mouse anti-Rac1 antibody (Cell Signaling, USA) and HRP-conjugated secondary antibody. Activated Rac1 was detected using an ECL kit.

### Quantitative Real-time Polymerase Chain Reaction (qRT-PCR)

Total RNA was isolated with TRIzol reagent kit (Invitrogen, CA, USA). cDNA was synthesized from 2 μg of total RNA by using Script M-MLV (Tiangen Biochemical Technology, China). Real-time polymerase chain reaction (PCR) used 2×SuperReal PreMix Plus (SYBR Green, Tiangen Biochemical Technology, China) and 50 × ROX Reference Dye on ABI7500 QPCR System. SuperReal PreMix Plus was activated by incubation at 95°C for 15 min, before 40 cycles of 95°C for 10 s, 58°C for 30 s, and 72°C for 32 s. Fluorescence was measured using ABI7500 after the 72°C extension step. The samples were run in triplicate. β-actin was used as an internal control. A melting-point dissociation curve generated by the instrument was used to confirm that only a single product was present. Quantification of relative gene expression was calculated by the comparative Ct method (2-ΔΔCT). Results were expressed as relative to control. PCR primers used for the amplification of TLR4 and β-actin mRNAs were designed by Invitrogen.

TLR4 forward primer: 5′ CCTTAAAGAAACTAAATGTGGCTCA 3′

Reverse primer: 5′ TCTAAAGAGAGATTGACTTGGGGAT 3′

β-actin forward primer: 5′ CCCATCTATGAGGGTTACGC 3′

Reverse primer: 5′ TTTAATGTCACGCACGATTTC 3′.

### Statistical Analysis

Data were expressed as means±SEM. Statistical evaluation used one way ANOVA by GraphPad Prism 5.0, and P<0.05 was considered to be significant.

## Results

### 1. LPS Stimulates VSMCs Proliferation, and can be Partly Blocked by Pre-incubation with LY294002 or NSC23766

**Figure 1:**
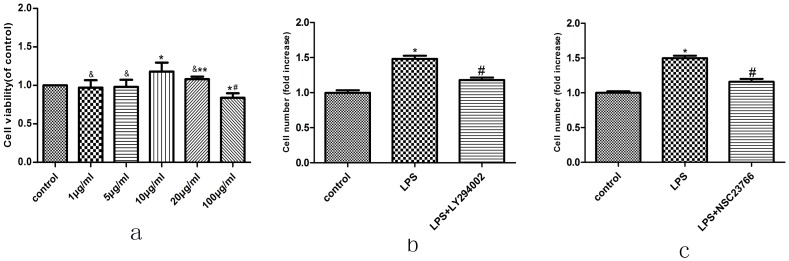
Effect of LPS on VSMC proliferation. (a) VSMCs were cultured in 96-well plates and incubated with 0, 1, 5, 10, 20 or 100 ug/ml concentration of LPS for 24 h. Then, the cell viability rate was determined by MTT assay as described in Materials and Methods section. Cell proliferation rate in the absence of treatment (control) was taken as 100%. (&compared with Control group, P>0.05; *compared with Control group, P<0.05; **compared with 10 ug/ml group, P>0.05; # compared with 10 ug/ml group, P<0.05). (b)(c)VSMCs were stimulated by 10 ug/ml LPS for 24 hours after 25 umol/L NSC23766 (Rac1 inhibitors) pretreatment 24 h or 10 umol/L LY294002 (Akt inhibitors) pretreatment 1 h, Cell proliferation was measured by cell counting. Data were obtained from three independent experiments and expressed as indicated. (*compared with Control group, P<0.05; # compared with LPS group, P<0.05).

To investigate the effect of LPS on proliferation, VSMCs were stimulated with different concentrations LPS for 24 h before MTT assay. Compared to the control group, LPS stimulated proliferation of VSMCs at 10 μg/ml (Fig. 1a). The number of VSMCs significantly increased after 10 μg/ml LPS treatment compared to the non-stimulated group, and the increased cells were reduced after pre-incubation in LY294002 (10 μmol/ml). Cell counts showed that LY294002 partially blocked LPS-stimulated VSMCs proliferation, indicating that Akt mediates VSMCs proliferation (Fig. 1b).

Rac1 is important in regulating cell proliferation of some cancer cell lines. Similarly, cell count assay showed that pre-incubation in NSC23766 (25 μmol/ml) partially blocked LPS-stimulated VSMCs proliferation, indicating that Rac1 mediates VSMCs proliferation (in Fig. 1c).

**Figure 2:**
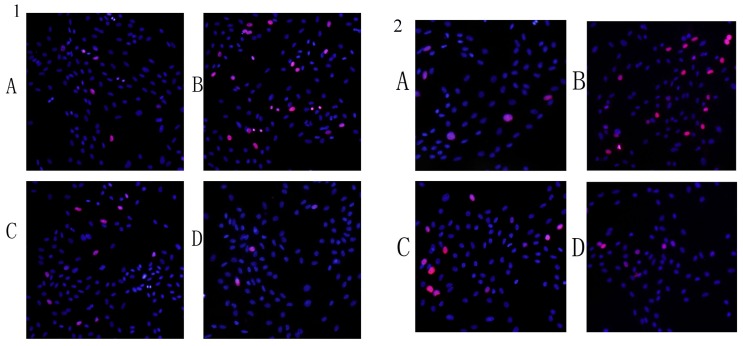
Detection of EdU incorporated into the DNA of cultured VSMCs by fluorescence microscopy. VSMCs were pretreated with NSC23766 (25 μmol/ml) 24 h or LY294002 (10 μg/ml) for 1 h before adding LPS (10 μ mol/ml). Each well was treated with 500 ul EdU (50 μmol/ml) and incubated for 2 h. After incubation, whole-cell extracts were prepared, and detected by EdU assay as described in Materials and Methods section.

EDU assay demonstrated that pre-incubation in LY294002(10 μmol/ml) or NSC23766 (25 μmol/ml) partially blocked LPS-stimulated VSMCs proliferation, indicating that Akt and Rac1 mediates VSMCs proliferation (Fig. 2).

### 2. LPS Induces Akt and Rac1 Activation and Increases TLR4 Protein Expression in VSMCs

**Figure 3:**
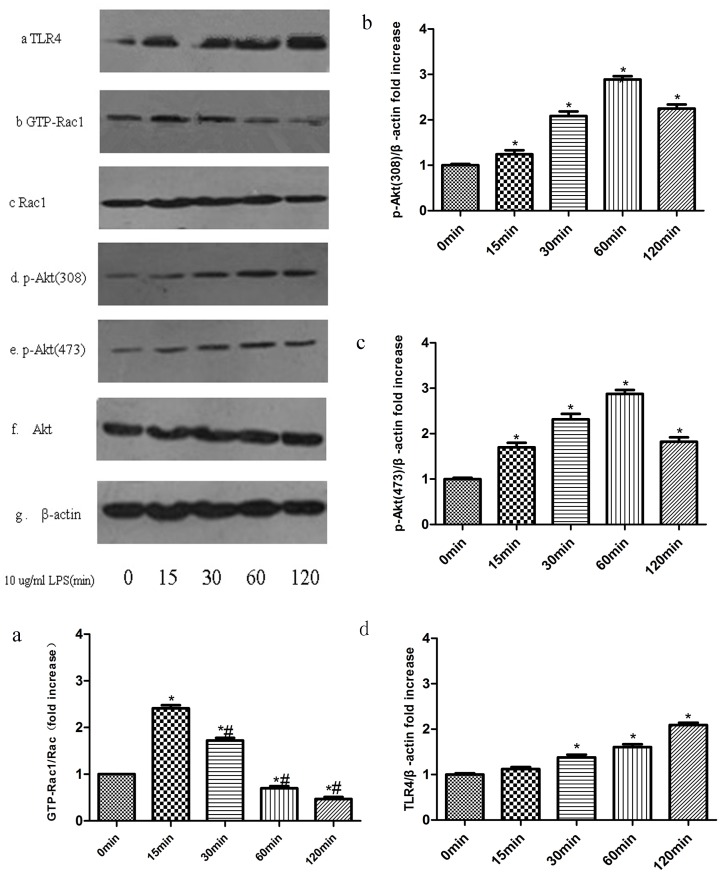
LPS Induces Akt and Rac1 activation and increased TLR4 Protein Expression in VSMCs. Rac1 activity was analyzed by GST pull-down methods (Figure 3a). Bar graphs show the relative intensity of each band (relative to that of total Rac1), which was measured by densitometry. The phosphorylation of Akt (Figure 3b and 3c) and the expression of TLR4 protein (Figure 3d) were analyzed by Western blotting. Bar graphs (Figure 3b, 3c, 3d) show the relative intensity of each band (relative to that of β-actin), which was measured by densitometry. Data represent the results of three independent experiments (means±SEM;*compared with Control group, P<0.05; # Compared with LPS (15 min) group, P<0.05).

Cells were lysed and protein was extracted from VSMCs after stimulation with 10 μg/ml LPS for 0, 15, 30, 60, or 120 min. Rac1 activity was measured with GST pull-down assays. There was transient activation of Rac1 after stimulation with 10 μg/ml LPS for 15 min (Fig. 3a). Expression of p-Akt (308) (Fig. 3b) and p-Akt (473) (Fig. 3c) protein levels were elevated and reached a maximum at 60 min after stimulation with LPS. Similarly, the expression of TLR4 protein increased and reached a maximum at 120 min after stimulation with LPS (Fig. 3d).

### 3. NSC23766 Inhibits LPS-induced Akt Phosphorylation

**Figure 4:**
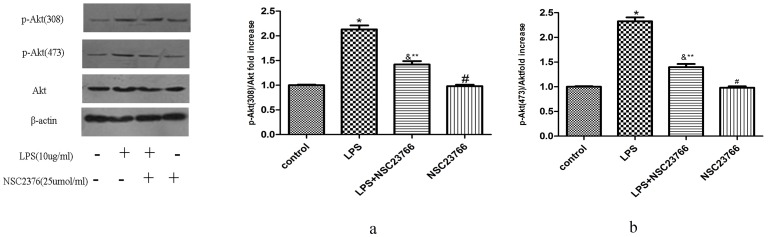
Role of Rac1 activity in LPS-induced Akt phosphorylation. VSMCs were stimulated by 10/ml LPS for 1 h after being pre-treated with 25 umol/L NSC23766 for 24 h and then the phosphorylation of Akt was analyzed by Western blotting. Bar graphs show the relative p-Akt intensity of each band (relative to that of Akt), which was measured by densitometry. Data represent the results of three independent experiments (means±SEM; *compared with Control group, P<0.05; &compared with Control group, P<0.05; **compared with LPS group, P<0.05, # compared with Control group, P>0.05).

To study the role of Rac1 in LPS-induced Akt activation, VSMCs were pretreated with 25 μmol/ml NSC23766 (Rac1 inhibitor) for 24 h before LPS (10 μg/ml) treatment, and p-Akt (308) and p-Akt (473) expression was analyzed by Western Blot. Pretreatment of VSMCs with NSC23766 partially inhibited LPS-induced p-Akt (308) (Fig. 4a) and p-Akt (473) (Fig. 4b) expression, indicating that Rac1 mediates p-Akt (308) and p-Akt (473) expression in VSMCs stimulated by LPS.

### 4. LPS-induced TLR4 Protein and mRNA Expressions is Mediated by Rac1and Akt Activation

**Figure 5:**
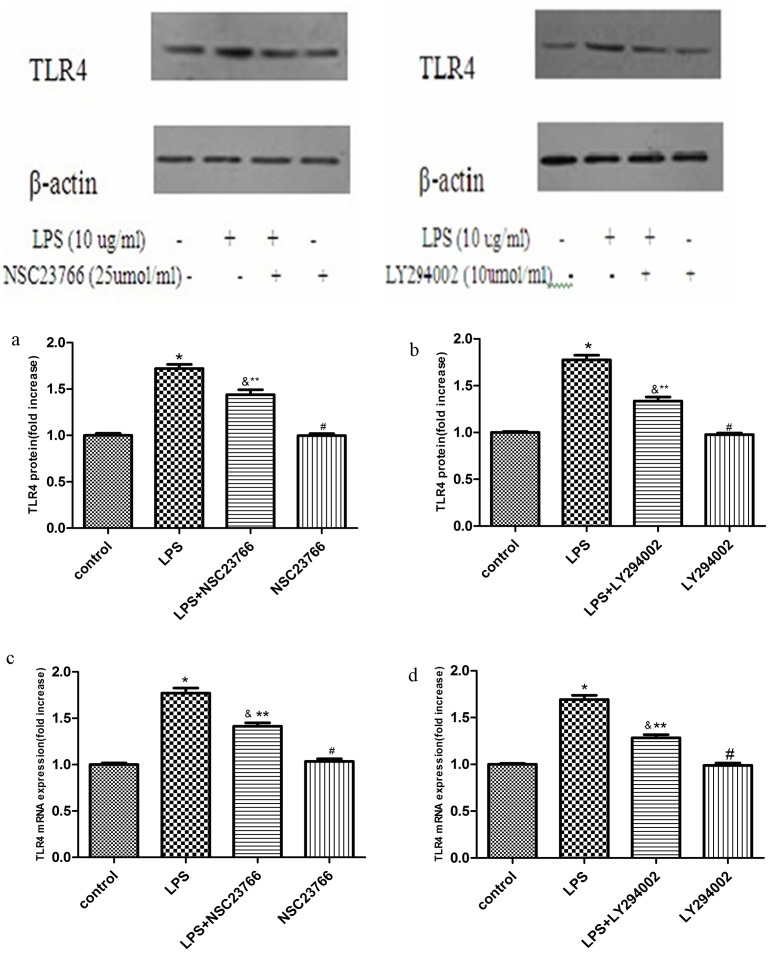
Role of Rac1 and Akt1 in LPS induced TLR4 expression. VSMCs were stimulated by 10/ml LPS for 2 h after being pretreated with 25 umol/L NSC23766 for 24 h (Figure 5a, 5c) or 10 umol/L LY294002 for 1 h (Figure 5b, 5d). Then the expression of TLR4 protein was analyzed by Western blotting and TLR4 mRNA analyzed by RT-PCR. Bar graphs show the relative intensity of each band (relative to that of β-actin), which was measured by densitometry. Data represent the results of three independent experiments # compared with Control group, P<0.05; *compared with LPS group, P<0.05).

To explore the role of Rac1 and Akt1 in LPS-induced TLR4 expression, VSMCs were pretreated with 25 μmol/ml NSC23766 for 24 h, or 10 μmol/ml LY294002, for 1 h before LPS treatment. TLR4 protein expression was analyzed by Western Blot. Pretreatment of NSC23766 or LY294002 significantly inhibited LPS-induced TLR4 protein expression (Fig. 5a and b), indicating that both Rac1 and Akt can mediate TLR4 protein expression.

Real-time PCR demonstrated that LPS-induced TLR4 mRNA expression was reduced by 25 μmol/ml NSC23766 (a Rac1 inhibitor) or 10 μmol/ml LY294002 (an Akt inhibitor) (Figure 5c and d). LPS-induced TLR4 mRNA expression was significantly reduced by NSC23766 or LY294002, suggesting that both Rac1 and Akt1 are also involved in LPS-induced TLR4 mRNA expression.

## Discussion

We have confirmed that LPS is a strong stimulator for VSMCs proliferation. The new findings concern the mechanism of LPS action on VSMCs proliferation, and the signaling pathway involved, which may be potential therapeutic targets for treating atherosclerosis. Rac1-mediated Akt activation is critical in LPS enhancement of TLR4 expression in VSMCs. The data provide evidence for the direct involvement of LPS-mediated VSMCs proliferation, which may contribute to the progression of cardiovascular disorders.

LPS initiated TLR4 receptor-mediated signaling pathway is of pivotal importance in vascular remodeling and neointimal formation after vascular injury [14]. Inhibiting the TLR4-mediated signaling pathway can prevent atherosclerosis developing, as has been verified in some in vitro studies [15]. In the carotid artery injury model, blocking TLR4 can reduce restenosis. Therefore, inhibition of LPS-mediated VSMCs proliferation and migration may a novel way of intervening in vascular hyperplastic disease [16]. However, the concrete mechanism of TLR signaling pathways in VSMCs proliferation and neointimal hyperplasia remain to be elucidated.

LPS is a strong stimulator of the pathogenesis of atherosclerosis [17]. LPS-induced TLR4 receptor activation involves many signal cascade reactions, including PI3K, mitogen-activated protein kinase (MAPKs), and IL-1R–associated kinases (IRAKs), all involved in cell proliferation and migration [18], [19]. IL-1R and TLRs share a common signaling pathway in leading NF-κB nuclear translocation involving myeloid differentiation protein 88 (MyD88), IRAK, tumor necrosis factor receptor-associated factor 6 (TRAF6) and IKKs [20], [21]. As an important factor for stimulating VSMCs proliferation, it is unclear how LPS influences TLR4 regulation of specific signaling pathways that promote VSMCs proliferation.

The Akt signaling pathway is associated with vascular inflammation, modulated by ROS [22]. Akt1 is downstream of PI3K signaling pathway, which is involved in many important intracellular physiological processes, including cell cycle regulation, survival, growth and migration [23]. Multiple growth factors through PI3K/Akt signal transduction pathway and Ser473 and Thr308 sites phosphorylation are necessary for Akt activation [24]. Akt activation is critical in the cellular responses associated with inflammatory stimuli, such as LPS and PDGF [25]. Hattori [26]reported that IFN/LPS promotes p-Akt expression in VSMCs. Lin reported that LPS induces TLR4 expression in VSMCs via MAPKs signaling pathways [27]. Whether LPS induces TLR4 expression in VSMCs via Akt needs further discussion.

More attention is now being paid to the role of the NADPH oxidase subunit in atherosclerotic lesions [28], [29]. Rac1, called the Ras related C3 botulinum toxin substrate1, belongs to the family of Rho-GTP enzymes located within the cell membrane. Rac1-regulated signaling can be mediated by a variety of downstream effectors, such as NF-κB [30]and HIF (hypoxia-inducible factor). Ferri reviewed the role of small GTPase protein Rac1 in cardiovascular diseases [31]; Rac-1 promotes pulmonary artery smooth muscle cell proliferation [32]. As a classic molecule for promoting cell proliferation and migration [33], [34], previous reports have shown that Rac1 initiates Akt activation, and has an important function in regulating proliferation and migration in other cell lines [35]. John reported that Rac1 participates in LPS activation of TLR4 expression [36]. In the carotid artery injury model, Ohkawara [37] reported that Rac1 activity is higher in atherosclerosis plaques, and is proportional to the degree of hardening of the arteries. Inhibition of Rac1 can inhibit VSMC proliferation induced by PDGF [38] and EGF [39]. Based on this information, inhibition of Rac1 presumably suppresses the proliferation of VSMCs induced by LPS. Indeed, our results show that LPS stimulates VSMCs proliferation, which can be partly blocked by pre-incubation with NSC23766.

PI3K can serve as an activator or effector of Rac1 in a stimulus- and cell-dependent manner [40], [41]. Both Akt and Rac1 are downstream of the LPS signal pathway [42], suggesting Rac1, Akt and TLR4 expression are heavily involved in VSMCs proliferation induced by LPS. p-Akt was partly inhibited by NSC23766. Other studies have shown that Rac1 mediates the pro-inflammatory phenotype of VSMCs, and that LPS treatment upregulates Rac1 expression, which potentially may be involved in vascular inflammation. In our work, VSMCs express p-Akt protein under basal conditions, which suggests that Rac1 has a function in LPS-induced p-Akt expression.

To explore the potential mechanisms in LPS-induced VSMCs proliferation, we studied the expression and activity of membrane-bound Rac1 activation, p-Akt (308) and p-Akt (473) expression and total TLR4 expression after LPS stimulation. Rac1 activity and p-Akt and TLR4 expression are gradually raised; Rac1 activation, and p-Akt (308), p-Akt (473) and TLR4 expression reached their highest peak at 15, 60 and 120 min, respectively. The activity time of the signal pathway suggests that p-Akt, TLR4 and GTP-Rac1 expression are involved in signaling cascades.

NSC23766 was used to confirm this regulation; thus the role of Rac1 in LPS-induced Akt phosphorylation was examined. Inhibition of Rac1 partially abolished p-Akt (308) and p-Akt (473) expression. Other studies have suggested that TLR4 mediates the pro-inflammatory phenotype of VSMCs, and that TLR4 expression is involved in vascular inflammation [43], [44]. To explore the role of Rac1and Akt in LPS-induced TLR4 expression, NSC23766 and LY294002 were given as pretreatments. VSMCs displayed a constitutive low-level expression of TLR4 protein under basal conditions. LPS-induced TLR4 expression in VSMCs could be partially blocked by pre-incubation NSC23766 or LY294002, which shows that both Rac1 and Akt are involved in LPS-induced TLR4 expression. The results also show that the difference in the expression of TLR 4 in LPS stimulation after NSC23766 or LY294002 pretreatment groups has statistical significance compared with control group. Thus there may be other mechanisms involved in the regulation of TLR4 expression in VSMCs.

**Figure 6:**
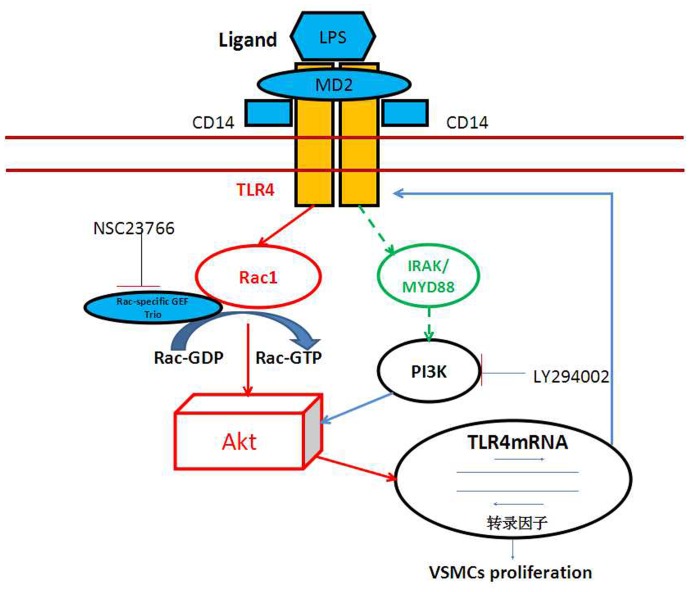
LPS stimulates VSMCs proliferation via TLR4/Rac1/Akt signaling pathway, and there is positive feedback. (The dotted line means TLR4 may activate other molecules such as IRAK, MYD88, and then activating PI3K).

In summary, LPS-initiated TLR4 promotes rat aortic SMC proliferation, and inhibition of Rac1 or Akt activation can inhibit rat aortic SMC proliferation induced by LPS, respectively. LPS promotes Rac1 activation, upregulates p-Akt expression and increases TLR4 expression in rat aortic VSMCs. Rac1 mediates p-Akt (308) and p-Akt (473) protein expression that have been stimulated by LPS. Inhibition of Rac1 or Akt activation can reduce TLR4 expression induced by LPS. Our findings suggest that LPS promotes rat aortic SMC proliferation associated with cardiovascular diseases, and a positive feedback regulation of VSMCs proliferation is mediated through TLR4/Rac1/Akt signaling pathway (Fig. 6).

However, our experiments have some limitations. Although it has been reported that deficiency of TLR4 is associated with reduced atherosclerosis in mice and attenuated pro-inflammatory state in diabetic mice, it remains unknown whether treatment with TLR4 antagonists reduces atherosclerosis in non-diabetic and diabetic mice [45]. We did not examine whether LPS administration affects the expression of TLR4 in vivo. It is unknown whether downregulation of TLR4 can prevents the effect of LPS on VSMCs proliferation. It is also unknown whether LPS promotes VSMCs migration and possesses the same positive feedback regulation through the TLR4/Rac1/Akt pathway. Others have begun pay attention to the regulatory role of Rac1 in in cardiovascular disease. However, the relationship between Rac1 and TLR signaling pathways is rarely explored. Our experiments indicate LPS induces Rac1 activation, and Rac1 mediates TLR4 expression. How Rac1 is activated by TLRs needs further examination. We have not explored other TLR4 signaling pathways involving MyD88 or TRIF. Whether Rac1 activation following LPS treatment depends on MyD88 or TRIF requires further investigation [46]. We have not analyzed chemokine or cytokine expression, such as IL-6 or IL-1 alpha, which promote a proliferative and pro-inflammatory phenotype in human VSMCs [47]. Whether autocrine or paracrine mechanisms are involved in the activation of Rac1 by LPS/TLR4 signaling in VSMCs is unknown. Without using a TLR4 inhibitor or RNAi-mediated knock-down of TLR4, TLR4 independent effects caused by LPS remain unknown. Thus the TLR4 specificity of the increased proliferation seen after incubation of VSMCs with LPS could be verified. In all, the role of Rac1 in the TLR-signaling network requires further investigation.
